# A *Hylarana latouchii* Skin Secretion-Derived Novel Bombesin-Related Pentadecapeptide (Ranatensin-HLa) Evoke Myotropic Effects on the in vitro Rat Smooth Muscles

**DOI:** 10.3390/toxins11040204

**Published:** 2019-04-05

**Authors:** Yan Lin, Nan Hu, Haoyang He, Chengbang Ma, Mei Zhou, Lei Wang, Tianbao Chen

**Affiliations:** 1 College of Bee Science, Fujian Agriculture and Forestry University, Fuzhou 350002, China; 2 Natural Drug Discovery Group, School of Pharmacy, Queen’s University, Belfast BT9 7BL, Northern Ireland, UK; nhu01@qub.ac.uk (N.H.); hhe06@qub.ac.uk (H.H.); c.ma@qub.ac.uk (C.M.); m.zhou@qub.ac.uk (M.Z.); l.wang@qub.ac.uk (L.W.); t.chen@qub.ac.uk (T.C.)

**Keywords:** frog, mass spectrometry, molecular cloning, bombesin-related peptide, smooth muscle

## Abstract

Amphibians have developed successful defensive strategies for combating predators and invasive microorganisms encountered in their broad range of environments, which involve secretion of complex cocktails of noxious, toxic and diverse bioactive molecules from the skins. In recent years, amphibian skin secretions have been considered as an extraordinary warehouse for the discovery of therapeutic medicines. In this study, through bioactivity screening of the *Hylarana latouchii* skin secretion-derived fractions, a novel peptide belonging to ranatensin subfamily (ranatensin-HLa) was discovered, and structurally and pharmacologically-characterised. It consists of 15 amino acid residues, pGlu-NGDRAPQWAVGHFM-NH_2_, and its synthetic replicate was found to exhibit pharmacological activities on increasing the contraction of the in vitro rat bladder and uterus smooth muscles. Corresponding characteristic sigmoidal dose-response curves with EC_50_ values of 7.1 nM and 5.5 nM were produced, respectively, in bladder and uterus. Moreover, the precursor of ranatensin-HLa showed a high degree of similarity to those of bombesin-like peptides from *Odorrana grahami* and *Odorrana schmackeri*. *Hylarana latouchii* skin continues to serve as a storehouse with diverse lead compounds for the development of therapeutically effective medicines.

## 1. Introduction

The word “amphibian”, *amphi* meaning both and *bios* meaning life, tells us much about the amphibian lifestyle—a double life. More specifically, amphibians are a class of animals that many of them have a biphasic lifestyle, undergoing shift from strictly aquatic larvae to more terrestrial adults. They are widespread on the majority of continents apart from Antarctica [1,2]. In the present day, there are 7975 species of amphibians in total within the Class Amphibia, with 88% of anurans (frogs and toads) [3]. From aquatically to terrestrially, amphibians have gone through drastic environmental changes, in which the highly-specialised skin plays a multitude of vital roles in their adaptability, including respiration, thermoregulation, water absorption, camouflage, defensive barrier, and osmoregulation [4]. Amphibian skin is characterised by numerous dispersed mucous glands which excrete mucus continually to maintain the moisture of skin, and granular glands that discharge extremely toxic components to ward off predators and pathologic microorganisms [5,6].

Amphibian skin secretions contain a very high level of diversity of biochemical compounds such as bioactive peptides/proteins, biogenic amines, steroids and alkaloids, possessing various pharmacological properties like hallucinogenic, cardiotonic, analgesic, antibacterial, antifungal, antidiabetic and antineoplastic activities [7,8,9]. Peptides constitute the most significant molecular group in the secretions of frog skin, and can be broadly classified into two main groups—antimicrobial peptides and pharmacologically-active peptides [8,10]. The former group of peptides are the fundamental elements of frogs’ innate immune system against pathogen attack, serving as the front line of an anti-infective defence barrier [11]; the second group of peptides execute irreplaceable responsibilities of regulating the physiological functions of the skin itself [12] and disrupting the homeostatic balance of predators [13]. Since analogues of a number of amphibian skin-derived peptides can be found in mammalian gastrointestinal tract, nervous tissues and endocrine organs but exist in extremely high quantities in amphibian skin, they have excited the interest of scientists in developing novel therapeutic drugs and elucidating relevant physiological processes in mammals [10,14].

Bombesin, identified in the skin of *Bombina bombina* [15], is one of the most important neuropeptides, whose discovery promoted the isolation of two counterparts in mammals—gastrin-releasing peptide (GRP) and neuromedin B (NMB) [16,17]. Bombesin-like peptides exhibit numerous pharmacological functions, such as stimulation of vascular and extra vascular smooth muscle contraction, renal circulation and gastric secretion [18]. They can be divided into three subfamilies: bombesin, ranatensin/litorin and phyllolitorin [19,20]; each member of them has a common structure in the C-terminal tetrapeptide amide. For bombesins, the final four amino acids are -Gly-His-Leu-Met-NH_2_; -Gly-His-Phe-Met-NH_2_ is at the C-termini of ranatensin/litorin subfamily; and phyllolitorin subfamily ends with -Gly-Ser-Phe/Leu-Met-NH_2_ [21,22]. They function by binding to five closely-related bombesin-like G-protein coupled receptors, including the NMB receptor (BB1-R), the GRP receptor (BB2-R), the bombesin receptor subtype-3 (BB3-R), the bombesin receptor subtype-4 (BB4-R) and the bombesin receptor subtype-3.5 (BB3.5-R) [21,23,24,25].

Here, from the skin secretion of the broad-folded frog, *Hylarana latouchii*, a structurally novel ranatensin-related peptide was isolated and investigated for its pharmacological activity. It evoked contractile effects on rat bladder and uterus by binding to NMB receptor and GRP receptor in the in vitro smooth muscle assays. Furthermore, prepropeptides of ranatensin-HLa and other closely related peptides including previously discovered ranatensin-HL were analysed to illustrate the evolutionary strategies of some species in the Ranidae family.

## 2. Results

### 2.1. Bioactivity Screening Resulted in Discovery of Ranatensin-HLa

**Figure 1:**
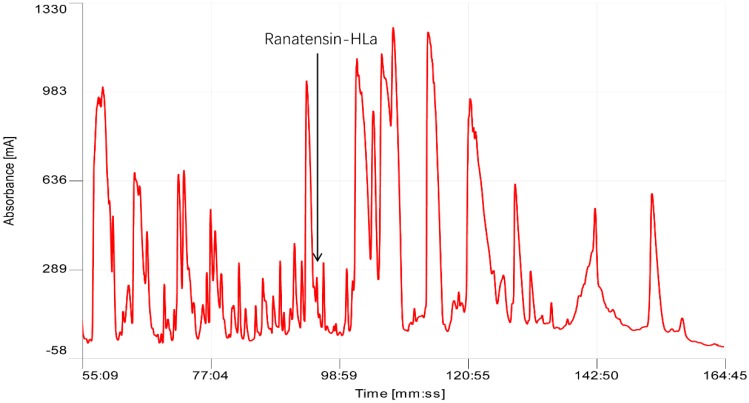
Chromatogram of *Hylarana latouchii* skin secretion indicating myoactive peptide (ranatensin-HLa). Ranatensin-HLa was eluted at around 93 min as pointed with an arrow.

**Figure 2:**
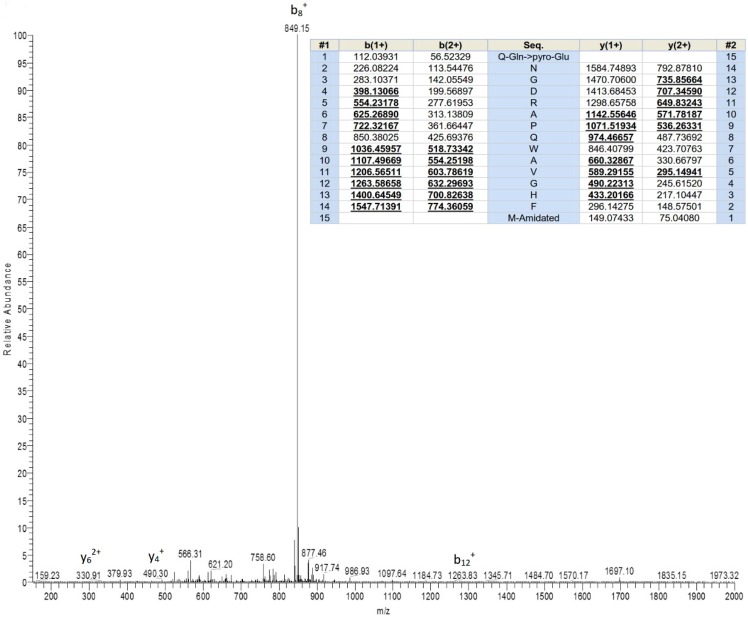
Mass spectrum of the primary structural analysis of the HPLC fraction possessing myotropic activity using MS/MS fragmentation sequencing. Predicted *b*- and *y*- MS/MS fragment ion series (both singly- and doubly-charged) are shown in panel. Observed ions are indicated in bold underlined typeface.

**Figure 3:**
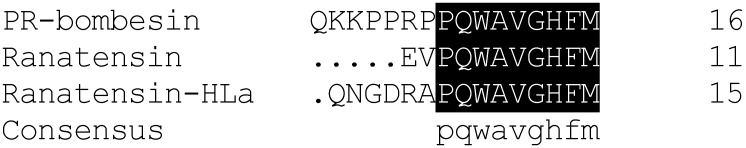
Sequences alignment of the mature peptides with the highest similarity in the bioinformatic analysis of ranatensin-HLa through inquiring NCBI-BLASTp database. Ranatensin-HLa and a bombesin-related peptide (PR-bombesin) (*Bombina maxima*) (Accession number: AAM10624.1) showed 62% identity. Meanwhile, Ranatensin-HLa and ranatensin (*Rana pipiens*) (Accession number: 701177B) showed 60% identity.

Following chromatographic fractionation of *H. latouchii* skin secretion, the bioactivity screening of sequential fractions resulted in the authentication of a component in fraction # 93 possessing considerable myoactivities towards rat urinary bladder and uterus. The active fraction was in accord with an absorbance peak in chromatogram with retention time of about 93 min as shown in Figure 1. Matrix-assisted laser desorption/ionisation time-of-flight (MALDI-TOF) mass spectrometric analysis indicated that this fraction comprised a single predominant peptide with molecular weight of 1695.32 Da. Its primary structure was further analysed by MS/MS fragmentation sequencing (Figure 2), which was followed by structural bioinformatic analysis inquiring the National Centre for Biotechnology Information-Basic Local Alignment Search Tool Protein Database (NCBI-BLASTp), indicating the highest sequence similarity with a proline rich bombesin-related peptide (PR-bombesin) from *Bombina maxima* and ranatensin from *Rana pipiens*. The sequence alignment of mature PR-bombesin, ranatensin and ranatensin-HLa was shown in Figure 3. Due to the identity of the C-terminal 4 residues (-Gly-His-Phe-Met-NH_2_) with the typical ranatensin subfamily peptide, this peptide was regarded as a member of the ranatensin group and was assigned a name of ranatensin-HLa (“HL” represented *Hylarana latouchii* and “a” represented distinction from a previously described peptide named ranatensin-HL).

### 2.2. Molecular Cloning of the cDNA Encoding the Biosynthetic Precursor of Ranatensin-HLa

**Figure 4:**
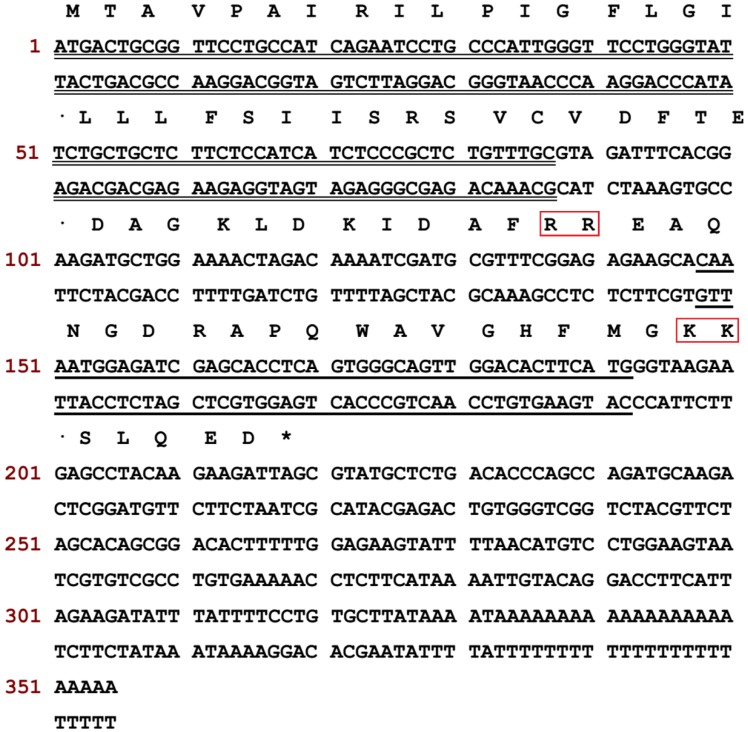
Nucleotide and amino acid sequences of the cDNA encoding the prepropeptide of ranatensin-HLa. Putative signal peptide domain is double-underlined, mature peptide sequence is single-underlined and stop codon is marked by an asterisk. The constructive convertase processing sites for the release of mature peptide are squared in red.

**Figure 5:**
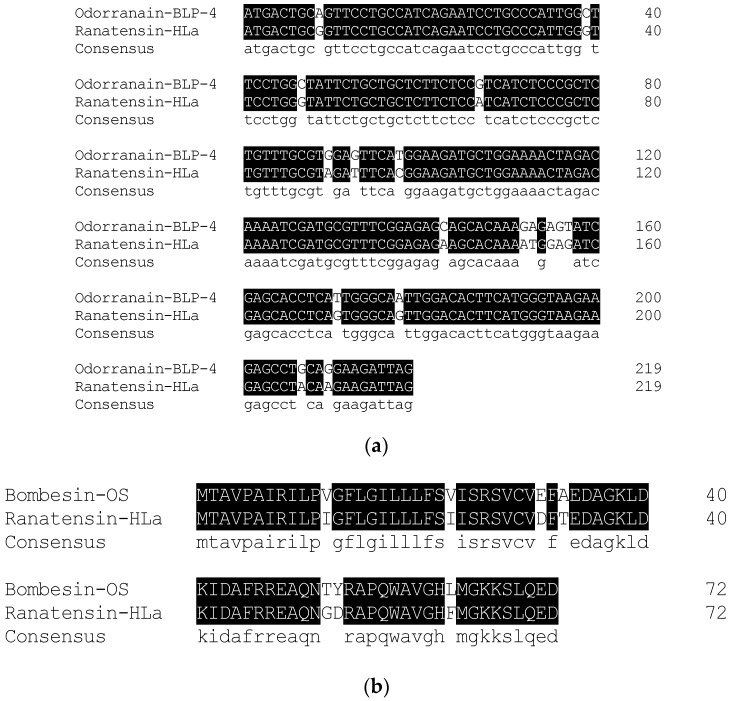
Sequence alignments of the full-length open-reading frames of prepro-ranatensin-HLa and the precursors with the highest sequence homology in the bioinformatic analysis using NCBI-BLAST nucleotide and protein database. (**a**) Ranatensin-HLa ORF and a bombesin-like peptide (odorranain-BLP-4) precursor (*Odorrana grahami*) (Accession number: DQ836112.1) showed 92% of sequence identity; (**b**) Ranatensin-HLa ORF and a bombesin (bombesin-OS) precursor (*Odorrana schmackeri*) (Accession number: ATP61827.1) showed 90% of sequence identity.

According to the primary structure of ranatensin-HLa, a degenerate primer was designed and used to clone the complete cDNA encoding the prepropeptide of ranatensin-HLa. Consequently, the full-length cDNA was iteratively cloned from the cDNA library established from *H. latouchii* skin secretion through RACE PCR. The translated amino acid sequence of the open-reading frame (ORF) of the precursor was composed of 72 residues which was constituted by a canonical architecture involving a putative signal peptide domain, an N-terminal spacer peptide domain flanked by a typical constructive dibasic amino acid cleavage site (-RR-), a single copy of mature peptide (15 amino acids) and a short C-terminal extended peptide containing another convertase cleavage site (-KK-) and a amide donor (glycine) (Figure 4). The sequence of ranatensin-HLa open-reading frame was analysed by searching for the nucleotide and protein database within NCBI-BLAST, indicating significant sequence homology with bombesin-like peptide precursors identified in *Odorrana grahami* (odorranain-BLP-4) and *Odorrana schmackeri* (bombesin-OS), respectively. The corresponding open-reading frame nucleotide and amino acid sequences alignments were shown in Figure 5. The cDNA sequence of ranatensin-HLa precursor with accession code LR032001 has been deposited in the EMBL Nucleotide Sequence Database.

### 2.3. Pharmacological Effects of Ranatensin-HLa on Rat Smooth Muscles

**Figure 6:**
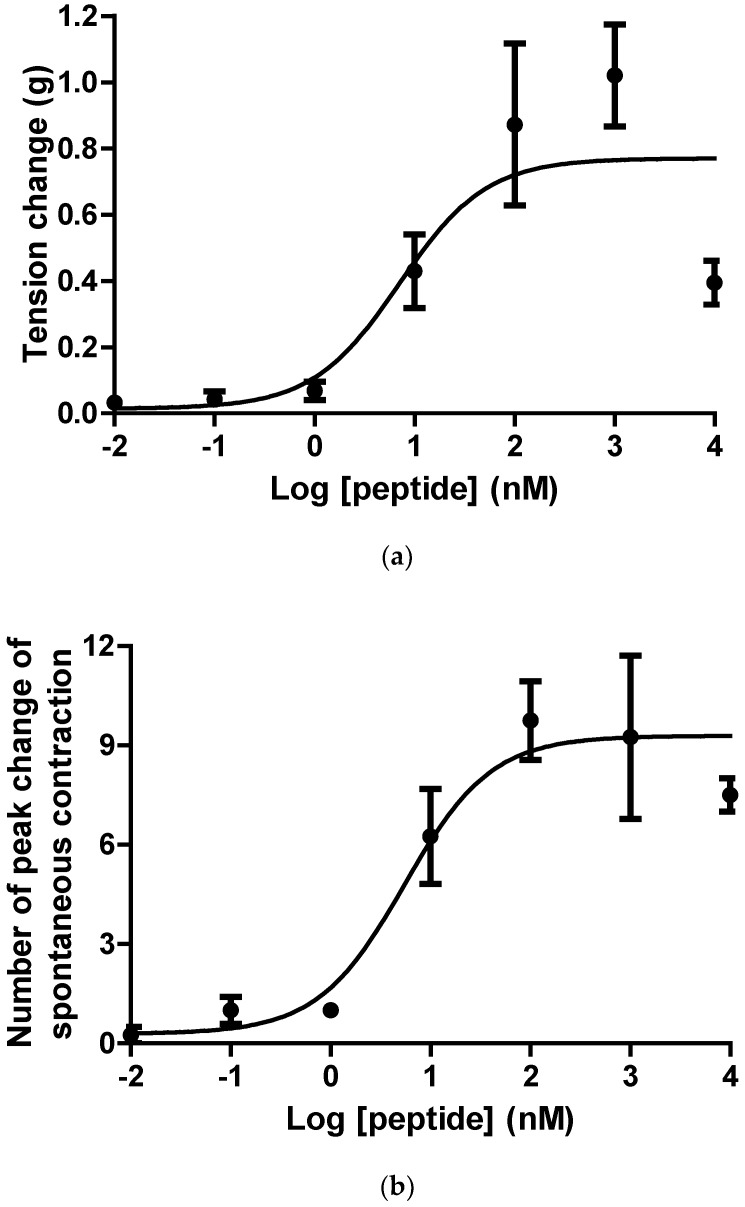
Myotropic effects of synthetic ranatensin-HLa on isolated smooth muscle preparations from rat bladder and uterus. (**a**) Dose-response curve of the effectiveness of ranatensin-HLa on bladder smooth muscles (EC_50_ = 7.1 nM); (**b**) Dose-response curve of the effectiveness of ranatensin-HLa on uterus smooth muscles (EC_50_ = 5.5 nM). Five determinations were conducted for the generation of each data point.

The purified synthetic replicates of ranatensin-HLa were used to assess the biological activities on isolated bladder and uterus muscle strips, in which it exhibited pacific pharmacological activities on stimulating contraction of bladder and enhancing the periodicity of spontaneously contractive activity of uterus with EC_50_ values of 7.1 nM and 5.5 nM in a dose-dependent manner (Figure 6).

## 3. Discussion

In recent years, natural drug discovery has been intensified and extended to structurally- and functionally-diverse compounds of amphibian origin. In reality, since ancient times, amphibian-derived substances have been applied for many conditions; for instance, the skin secretion of poison-dart frogs have been used for the purpose of effective hunting by Native Americans up to the present [26,27], and Chan Su, processed from the dried skin secretion of *Bufo bufo gargarizans* Cantor or *Bufo melanostictus* Schneider with the efficacy of detoxification, has been an important traditional remedy for around 3000 years [28,29,30,31]. With the advancement in the technologies of isolation, identification and analysis, it has been recognised that the skin of amphibians is an extraordinary arsenal of a plethora of biologically-active components possessing diverse pharmacological properties [7,8,9]. These bioactive compounds are endogenously produced and deposited in granular glands, being discharged to the surface of integument upon stimulation to implement diverse physiological and defensive functions [14,32].

**Figure 7:**
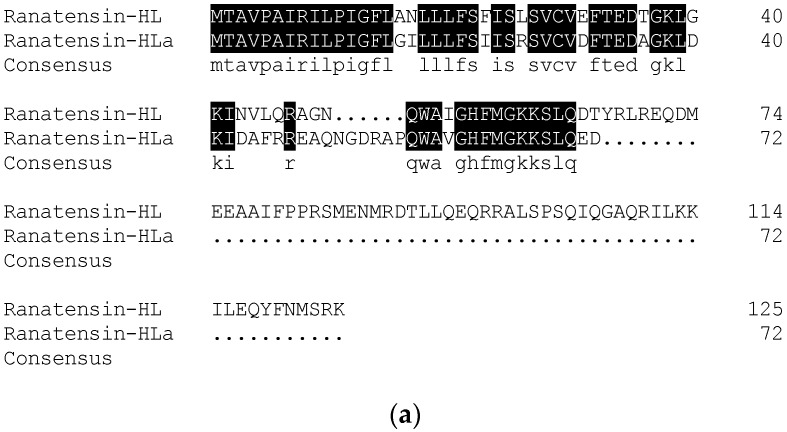

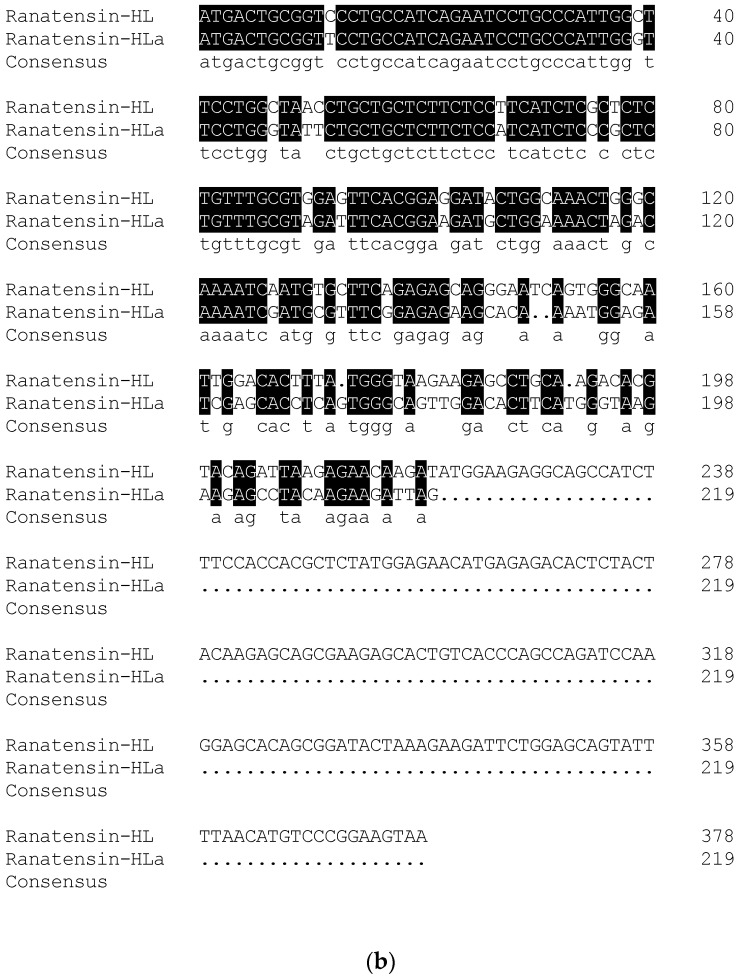
Comparison of the nucleotide and amino acid sequences of the biosynthetic precursors of ranatensin-HL [33] and ranatensin-HLa. (**a**) Comparison of the translated amino acid sequences of ranatensin-HL and ranatensin-HLa precursors; (**b**) Nucleotide sequences comparison of ranatensin-HL and ranatensin-HLa precursors.

In this study, another bombesin-like peptide termed ranatensin-HLa with new structure and belonged to ranatensin subfamily was isolated from the skin secretion of *H. latouchii* following the discovery of ranatensin-HL in 2017. The overall structure of ranatensin-HLa precursor was consistent with other prepropeptides from frog skin, containing a characteristic construction of “signal peptide--N-terminal spacer peptide--mature peptide”. Besides, as other neuropeptides from amphibian skin, including ranatensin-HL, the N-terminal glutamine of ranatensin-HLa was also converted into pyroglutamine, and it was also C-terminally amidated. Nevertheless, compared with ranatensin-HL which was released peculiarly from a 125-amino acid residue large precursor, the size of ranatensin-HLa precursor (72 amino acid residues) was similar to that of other ranid frogs-derived bombesin-related peptide precursors [33]. Moreover, like most biosynthetic precursors of bombesin-related peptide formerly cloned from Ranidae family, yet unlike ranatensin-HL precursor, the N-terminal of ranatensin-HLa was flanked by a common convertase proteolytic site made up of dibasic amino acids (Arg-Arg) (Figure 4). According to the sequence comparison of the nucleotides and amino acids of the open-reading frames of ranatensin-HL and ranatensin-HLa (Figure 7), the signal peptides and N-terminal spacer peptides were highly conserved, while the mature peptides were significantly distinct, implying that they might originate from a mutual ancestral gene and became C-terminally different due to the stress response to the threats encountered in which the functional region should evolve rapidly in order to overcome different predators and microorganisms existing in a certain ecological niche. It has been speculated that such diverse molecules in a single species may result from the associated effects of gene duplications, mutation and positive selection [34,35,36]. Consequently, it makes predators or pathogens more difficult to fight against the components in such complicated cocktails of skin secretions and protects frogs from predators’ attack or invasive microorganisms to some extent.

The nucleotide and amino acid sequences of the biosynthetic precursor of ranatensin-HLa were highly homological to those of bombesin-like peptides from *Odorrana grahami* (odorranain-BLP-4) and *Odorrana schmackeri* (bombesin-OS) (Figure 5). Additionally, taking the high homology of ranatensin-HL from *H. latouchii* and another bombesin-related peptide from *Lithobates catesbeianus* [33] into consideration, we guessed that *H. latouchii*, *Odorrana graham*, *Odorrana schmackeri* and *Lithobates catesbeianus* were likely to have retained bombesin/ranatensin peptides in their skin secretion, or these peptides might be the outcome of convergent evolution [37].

**Figure 8:**
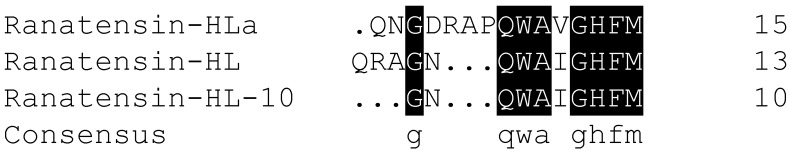
Comparison of ranatensin-HLs on the primary structures [33].

In terms of the pharmacological activity of ranatensin-HLa, it stimulated similar contractile frequency in rat uterus as ranatensin-HL and was more potent in evoking rat bladder contraction (Table 1). Since NMB- and GRP-preferring subtype receptors are expressed in bladder and uterus respectively [38,39], it was speculated that both ranatensin-HL and ranatensin-HLa might cause myotropic contraction by binding to the NMB receptor in bladder and GRP receptor in uterus. The greater potency of ranatensin-HLa towards bladder may be attributed to either the length or the composition of the N-terminal amino acids (Figure 8). Furthermore, as both ranatensin-HL and ranatensin-HLa were more effective than ranatensin-HL-10 in tested smooth muscles, it further suggested that the longer stretch of N-terminal might be beneficial to the efficacy of peptides [40] in exposing functional cores or extending half-life [41] (Figure 8 and Table 1).

Undoubtedly, frog skin will continue to serve as a treasury with extraordinarily-diverse lead compounds for the development of therapeutically effective medicines. Moreover, the dissimilar bioactive peptides present in different species and the corresponding precursor-encoding cDNA sequences will offer an additional insight into the evolutionary relationships between different specie [32,42].

## 4. Conclusions

Through the combination of mass spectrometry and molecular cloning, another naturally-occurring bombesin-related peptide belonging to ranatensin subfamily was characterised in *H. latouchii* skin secretion. In the in vitro pharmacological investigation, it displayed moderate myoactivity on rat bladder and uterus. The precursor of ranatensin-HLa was compared with that of ranatensin-HL previously discovered from *H. latouchii*; as a result, the signal peptides and N-terminal spacer peptides were highly conserved, but remarkable mature peptide modification was observed, which provided the evidence that the diversity of bioactive substances within a single species might be ascribed to selective pressure and might eventually lead to the frogs’ optimisation of adaptation.

## 5. Materials and Methods

### 5.1. Specimen Biodata and Skin Secretion Acquisition

Adults of the broad-folded Frog, *Hylarana latouchii* were captured in Fujian Province of the People’s Republic of China. Frogs were stimulated by mild electricity on the dorsal skin surface to discharge the defensive skin secretion which was subsequently rinsed off with distilled deionised water to be collected. The detailed methods were previously described [43]. The collected skin secretion was then lyophilised and kept at −20 °C prior to usage. The procedure of secretion acquisition had been overseen by the Institutional Animal Care and Use Committee (IACUC) of Queen’s University Belfast, and approved on 1 March 2011. It was carried out under the UK animal (Scientific Procedures) Act 1986, Project licence PPL 2694, which was issued by the Department of Health, Social Services and Public Safety, Northern Ireland.

### 5.2. Chromatographic Fractionation of H. latouchii Skin Secretion with Reverse Phase HPLC

A sample made from dissolving 5 mg of lyophilised *H. latouchii* skin secretion in 1 mL of trifluoroacetic acid (TFA)/water (0.05/99.95, *v*/*v*) was subjected to reverse phase HPLC chromatography. It was clarified and loaded onto a Jupiter C-5 semi preparative column (300 Å, 5 µm, 25 cm × 1 cm, Phenomenex, Macclesfield, Cheshire, UK) which was installed in a Cecil Adept CE4200 HPLC system (Amersham Biosciences, Buckinghamshire, UK) equipped with a Powerstream HPLC software for chromatographic fractionation. Chromatography was performed by eluting from 0.05/99.95 (*v*/*v*) TFA/water to 0.05/19.95/80.00 (*v*/*v*/*v*) TFA/water/acetonitrile over 240 min at a flow rate of 1 mL/min. The effluent fractions were detected at λ214 nm, and were collected automatically and continuously every minute. One hundred microliter of each collected fraction was transferred to be freeze-dried and stored at −20 °C for the subsequent screening of myoactivity employing smooth muscle bioassays.

### 5.3. Myoactivity Screening of Chromatographic Fractions

According to the institutional animal experimentation ethics and UK animal research guidelines, the method of carbon dioxide asphyxiation in combination of cervical dislocation was employed to realize euthanasia of female Wister rats with weights of 250-300 g for the in vitro smooth muscle pharmacological assays. Upon removal of the intact urinary bladder, uterine horns, ileum and proximal rat tail artery from each rat by dissection, they were kept in ice-cold Kreb’s solution (118 mM NaCl, 4.7 mM KCl, 25 mM NaHCO_3_, 1.15 mM NaH_2_PO_4_, 2.5 mM CaCl_2_, 1.1 mM MgCl_2_ and 5.6 mM glucose) spiritedly pneumatic with 95% O_2_ and 5% CO_2_ immediately. Then, the dissected tissue strips were carefully mounted on a transducer and immersed in organ baths filled with flowing (2 mL/min) Kreb’s solution constantly bubbled with mixed 95% O_2_ and 5% CO_2_ at 37 °C. Prior to the application of sample solutions, the tissue preparations were allowed to be equilibrated under the normal physiological tension of 1.0 g (for bladder) and 0.5 g (for bladder, uterus, artery and ileum) for 1 h. Then, HPLC fractions dissolved in 22 μl of Kreb’s solution were added sequentially to the organ baths to examine the myotropic activity of each fraction.

### 5.4. Structural Characterization of Peptide Possessing Bioactivity

The molecular weight of the HPLC fraction which displayed myoactivity was primarily analysed using MALDI-TOF mass spectrometry (Voyager DE, PerSeptive Biosystems, Foster City, CA, USA). Then, the predominant peptide in the bioactive fraction was further fragmented in an LCQ-Fleet electrospray ion-trap mass spectrometer (Thermo Fisher Scientific, San Francisco, CA, USA) for MS/MS sequencing to determine the primary structure.

### 5.5. Molecular Cloning of the cDNA Encoding the Peptide Biosynthetic Precursor of Ranatensin-HLa

Based on primary structure analysis result, a degenerate sense primer (S: 5′-CARAAYGGNGAYMGNGCNCC-3′) complementary to pGlu-N-G-D-R-A-P- was designed. One milliliter of cell lysis/mRNA protection buffer provided by manufacturer (Dynal Biotec, Wirral, UK) was used to dissolve a total of 5 mg of lyophilised *H. latouchii* skin secretion, which was followed by polyadenylated mRNA isolation with magnetic oligo-dT beads (Dynal Biotech, Merseyside, UK). The isolated mRNA was then reverse transcribed to synthesise cDNA that was applied to run 3′-rapid amplification of cDNA ends (RACE) procedure with the designed degenerate primer in conjunction with a Nested Universal Primer (NUP) provided by kit (SMART-RACE kit, Clontech, Palo Alto, CA, USA) to get the 3′-end of biosynthetic precursor of myoactive peptide. The resultant PCR products were ligated into a pGEM-T easy vector (Promega Corporation, Southampton, UK) and sequenced using an ABI 3100 automated sequencer (Applied Biosystems, Foster City, CA, USA). The sequence information was used to further design a specific antisense primer (AS: 5′-GCATCTGGCTGGGTGTCAGAGCATA-3′) complementary to the 3′-end of the DNA sequence. Subsequently, this primer along with the NUP primer were used to conduct 5′-RACE PCR whose products were purified, cloned and sequenced as described above.

### 5.6. Fmoc Chemistry Solid-Phase Peptide Synthesis of Ranatensin-HLa

On the strength of results obtained from primary structure analysis and molecular cloning, the explicit sequence of the bioactive peptide could be established. It was followed by automatic synthesis using PS3 solid-phase peptide synthesiser (Protein Technologies, Tucson, AZ, USA) with the methodology of Fmoc chemistry. Reverse phase HPLC was employed for the purification of the synthetic products, and MALDI-TOF MS and MS/MS fragmentation sequencing were used for the confirmation of the purity and structural authenticity of the peptide.

### 5.7. The Effects of Synthetic Peptide on Rat Smooth Muscles Tension

Purified peptide was prepared in Kreb’s solution and diffused to the 2-mL organ baths with bladder and uterus tissue strips equilibrated under normal physiological tension to get final concentrations ranging from 10^−2^ to 10^4^ nM to produce dose-response curves. Between each dose, the smooth muscle preparations were subjected to periods of 10-min wash and 10-min equilibration. Tension changes of bladder tissue strips were magnified and recorded by pressure transducers hooked up to a PowerLab System (AD Instruments Pty Ltd., Oxford, UK), and the frequency changes of the spontaneous contraction of uterus were recorded by counting the contractile peaks. Peptide was investigated for the myoactivity using at least five muscle strips in each concentration.

### 5.8. Statistical Analysis

Data were analysed by GraphPad Prism 5 software (San Diego, CA, USA) to establish the EC_50_ values for the peptides and to generate dose-response curves featured by changes in tension (for bladder) or changes in contractive frequency (for uterus) against peptide concentrations using a “best-fit” algorithm. Data points represented mean values ± SEM from Student’s *t*-test.

## Figures and Tables

**Table 1:** Comparison of ranatensin-HLs on the myoactivities [33].

Peptides	Bladder (EC_50_, nM)	Uterus (EC_50_, nM)
Ranatensin-HLa	7.1	5.5
Ranatensin-HL	19.2	5.4
Ranatensin-HL-10	63.8	70.9
